# Neutrophil extracellular traps in fungal infections: A seesaw battle in hosts

**DOI:** 10.3389/fimmu.2022.977493

**Published:** 2022-09-14

**Authors:** Hua Zhong, Ren-Yi Lu, Yan Wang

**Affiliations:** School of Pharmacy, Second Military Medical University, Shanghai, China

**Keywords:** neutrophil, neutrophil extracellular traps, fungal infection, immunology, *Candida*, *Aspergillus*

## Abstract

Fungal infections are a growing health care challenge. Neutrophils play a key role in defense against fungal infections. There are many effective ways for neutrophils to eliminate fungal invaders, such as phagocytosis, oxidative bursts, and the formation of extracellular traps. This process has received considerable attention and has made rapid progress since neutrophil extracellular traps (NETs) formation was described. Here, we describe the formation, induction, and function of NETs, as well as fungal strategies against NETs hunting. We highlight the effects of NETs on common fungal pathogens and how these pathogens survive.

## Introduction

Fungal infections have long been a public health challenge. It mainly affects immunocompromised populations, such as solid organ transplant recipients and AIDS patients (1). Fungal co-infection has also been reported in COVID-19 patients during these years of pandemics (2). Among COVID-19 patients on mechanical ventilation in the ICU, fungal co-infection rates have been reported as high as 26.7% (3). Innate immune system plays an important role in defense against fungal infections. Mucosal barriers and chemicals work with natural killer cells and phagocytes. It is reviewed that *Aspergillus* species can interact with the innate immune system including macrophages, neutrophils, dendritic cells, and the complement system (4). These cells and proteins recognize and kill fungal pathogens, protecting our bodies from infection. Neutrophil (polymorphonuclear leukocyte), which is one kind of the phagocytes, plays a decisive role in this process (5). Invasive fungal diseases occur in up to 24% of patients with leukemia (6). Neutrophils can kill fungal pathogens by phagocytosis, production of reactive oxygen species (ROS) and formation of extracellular traps. Neutrophil extracellular traps (NETs) were first described as an antibacterial mechanism of innate immunity in 2004 when Volker Brinkmann *et al.* discovered that NETs could kill bacteria (7). In recent years, much progress has been made in the study of NETs, and the fungicidal effects of NETs have been described, such as the fungicidal effects of NETs on *Candida* spp. and *Aspergillus* spp. (8, 9). Rather than waiting to be killed, fungi have their own ways of fighting back. For example, *Aspergillus* spp. can invade the innate immune system by interfering with complement system and phagocytes [reviewed in (4)]. In this article, we focus on the interactions between NETs and several common fungal pathogens. The killing process of NETs against fungal pathogens and the strategies of pathogen resistance were reviewed. 

## Formation of NETs

NETs are fibrous three-dimensional network structures composed of nucleic acids and various granular proteins that neutrophils can release out of the cell in response to various stimuli. This structure traps pathogens such as bacteria and fungi, limits their spread through the body and kills them with high concentrations of toxic proteins. Other immune cells, such as eosinophils and mast cells, can also form similar structures, killing a variety of microorganisms and enhancing inflammatory immune responses (10, 11). NETs help eliminate pathogens, but excess NETs can cause damage to surrounding tissues either by themselves or by increasing the pro-inflammatory response. The generation of NETs is closely related to the occurrence and development of various diseases (12–14).

Since 2004, two major ways of releasing NETs have been identified, a) NETosis and b) rapid release of live neutrophils. NETosis is a classic way of releasing NETs, usually neutrophils release NETs by decondensation of chromatin resulting in cell death (15). Later, a novel way was discovered to form NETs by releasing mitochondrial DNA. This novel way of NETs formation does not require neutrophil death and therefore does not limit the lifespan of these cells (16).

### NETosis

NETosis begins with oxidative burst and activation of peptidyl arginine deiminase 4 (PAD4), which catalyzes the citrullination of arginine residues. The process leads to disassembly of nuclear envelope and chromatin decondensation (17). Chromatin then combine with neutrophil elastase (NE) and other cytoplasmic enzymes to form NETs. And NETs release upon plasma membrane rupture. The whole process takes about 4 hours and results in neutrophil death (15, 18–20). A recent study showed that intact F-actin dynamics and myosin II function are essential for the formation of NETs in response to different stimuli including *Candida albicans*. Neutrophils in patients with actin polymerization defects also failed to exhibit NETs, confirming this conclusion (21).

### Rapid release of live neutrophils

Live neutrophils can generate NETs under some stimuli. Interestingly, these NETs contain mitochondrial DNA instead of nuclear DNA (16). The process can be very quick since neutrophils expel mitochondrial DNA and assemble NETs outside the cell. Both this approach and the NETosis approach rely on ROS (22). There is also a ROS-independent fast-release mechanism without neutrophil death. In some Gram-positive bacterial infections, the nuclear membranes of neutrophils are separated and nuclear DNA is extruded out of the cell through vesicles. The anuclear neutrophils are still capable of migration and phagocytosis (23, 24).

## Induction of NETs in fungal infections

The induction of NETs is affected by a variety of inducing factors and the number of NETs produced is different. ROS (25), IL-8 (7), lipopolysaccharide (LPS) (7), complementary 5a (C5a) (16), phorbol-12-myristate-13-acetate (PMA) (7, 18), and glucose oxidase (GO) (25) are all inducers that can trigger the formation of NETs. And among them, ROS is one of the key factors that trigger the formation of NETs.

Fungal pathogens can also trigger the release of NETs. *C. albicans* is the most widely discussed fungal pathogen in the NETs release field (26). In addition, other *Candida* spp. (27, 28), *Aspergillus fumigatus* (8), *Histoplasma capsulatum* (29), *Phialophora verrucose* (30), *Paracoccidioides brasiliensis* (31), and *Scedosporium apiospermum* (32), have all been described as NETs release inducers. The common dermatophyte *Trichophyton rubrum* is also found to be a NETs inducer. Both conidia and hyphae of *T. rubrum* can induce NETs formation in a dose-dependent manner (33). *Cryptococcus neoformans* itself is not an inducing factor, but the capsular polysaccharide glucuronoxylomannogalactan (GXMGal) can induce the formation of NETs (34). Unfortunately, *Candida auris*, a recent emerging global public health threat, cannot induce the formation of NETs and is not effectively killed by neutrophils (35). Fungi come in a variety of forms, from small yeast to large hyphae and biofilms, which require neutrophils to respond in different ways to eliminate them. Both yeast and hyphal forms of *C. albicans* can activate NETs formation (26). However, the extracellular matrix of *C. albicans* biofilms does not trigger NETs, but instead impairs the NETs formation (36). Interestingly, recent studies have shown that the nucleic acids in the extracellular matrix of *C. albicans* biofilms can stimulate the release of NETs (37). A number of components in *C. albicans* have also been shown to stimulate NETs release, including dectin-2 (38), aspartic proteases, mannans, β-glucans (39), and farnesol (40).

## Function of NETs in fungal infections

NETs are mixtures of nucleic acids, histones, granular proteins, and cytoplasmic proteins, including NE, myelperoxidase (MPO), lysozyme C, and gelatinase (20). These components lead to the release of chemokines, the production of cytokines, the promotion of inflammatory disease and, of course, the killing of microorganisms (20). The mixture can trap fungi inside its 3D network structure and cause damage to fungi through the components it releases. In recent years, studies on the effects of NETs on *C. albicans*, *Aspergillus* spp., and *C. neoformans* have made progress (**Table 1**).

**Table 1:** Role of NETs in different fungal infections.

Species	Morphology	Antifungal activity of NETs	Reference
*C. albicans*	yeast	+++	(26)
	hyphae	+++	(26)
*A. fumigatus*	conidia	+	(41)
	hyphae	+	(41, 42)
*A. nidulans*	conidia	+	(43)
	hyphae	+	(43)
*C. neoformans*	yeast	+++	(34)
*T. rubrum*	conidia	++	(33)
	hyphae	+++	(33)
*C. auris*	yeast	–	(35)

+++, strong fungicidal activity; ++, moderate antifungal activity; +, weak inhibition activity; -, no antifungal activity.

### C. albicans

Opportunistic fungal pathogen *C. albicans* is a component of intestinal commensal microbiota that colonizes the intestines, skin, and oral mucosa of healthy humans (44). In immunocompromised populations, such as neutropenia patients, it can shift from colonization to invasion and spread in the body, causing systemic infection (45). *Candida* spp. are the most common pathogens of invasive fungal diseases (44, 46), in which *C. albicans* is the major cause for candidiasis (47). As a model organism, *C. albicans* is the first fungus to be shown to induce NETs *in vitro* (26).

On the one hand, the sensitivity of different morphologies of *C. albicans* to NETs immunoclearance is different. NETs have been shown to kill both yeast and hyphal forms of *C. albicans* (26). However, NETs cannot be produced upon *C. albicans* biofilms. Time-lapse imaging showed that neutrophils adhered only to hyphae and migrated on the biofilms (36). Another study showed that sub-inhibitory concentrations of echinocandins, an effective antibiofilm drug, promote the formation of NETs in *C. albicans* biofilms, including structures of DNA, histones, and antimicrobial proteins with antifungal activity (48). On the other hand, different isolates of *C. albicans* also modulate the function of NETs. By using a panel of clinical *C. albicans* strains, Madhu Shankar et al. found that the prototype strain SC5314 induced the most potent accumulation of ROS and NETs by neutrophils from all the isolates tested (49).

In response to microbial infections, neutrophils initiate NETosis *via* protein kinase C (PKC) and activate the nicotinamide adenine dinucleotide phosphate (NADPH) oxidase signaling cascade, leading to the accumulation of ROS (39, 50). Studies have shown that *C. albicans*-induced NETs production requires PKC, and PKC inhibitor Gö6976 can block this process (51). Another important component of NETs that kills fungi is calprotectin. Lack of calprotectin in NETs resulted in a complete loss of antifungal activity *in vitro* (52).

NETs also unmask *C. albicans* and make it expose immunogenic epitopes to the host. NETs trigger fungal cell wall remodeling and enhance immune recognition by Dectin-1 β-glucan receptors. This process involves fungal MAPK pathways, which dynamically relocalize cell wall remodeling machinery including Chs3, Phr1 and Sur7 (53).

### *Aspergillus* spp.

*Aspergillus* spp. are common spore-releasing environmental fungi. However, for immunocompromised individuals who are unable to adequately clear the spores from their lungs, they may develop invasive pulmonary aspergillosis (IPA), which is life-threatening (54). Two high-risk groups were patients with neutropenia or hematologic malignancy and patients with chronic granulomatosis (CGD) (55, 56).

Unlike *C. albicans*, *Aspergillus* spp. may be less susceptible to NETs. NETs did not kill either *A. fumigatus* or *A. nidulans* conidia (41, 57). They are more inclined to be engulfed by living neutrophils (8). Another study proves that adding DNase to neutrophils do not affect the killing efficiency of *Aspergillus* hyphae, which indicates that NETs formation does not contribute to this fungal killing process (42). NETs are more robust towards *A. fumigatus* hyphae than conidia, which is confirmed by both *in vitro* and *in vivo* experiments (8, 41).

Patients with CGDs usually exhibit deficient phagocyte NADPH oxidase function, which is essential in the formation of NETs (15). In a case report, CGD patients reconstructed the generation of NETs through gene therapy and restored neutrophil clearance of *A. nidulans* conidia and hyphae, which is associated with rapid cure of IPA (58). The authors soon verified this connection experimentally. Restoring NADPH function through gene complementation can restore the production of NETs *in vitro* (43). Further studies have shown that calprotectin plays a key role in human innate immunity against *Aspergillus* infection (43).

Some studies came to somewhat an opposite conclusion. On one hand, it is proved by confocal imaging that neutrophils from CGD patients can still form NETs under the stimulation of *Aspergillus* hyphae, although these cells with genetic immunodeficiencies have antifungal deficiency (42). The researchers also find that neutrophils from CGD patients cannot initiate NETs formation in response to PMA, which suggests different mechanisms between PMA and *Aspergillus* hyphae in inducing NETs formation (42). A relevant study shows that *Aspergillus* and β-glucan-induced NETs formation is regulated by PAD4 and CR3. The hyphae killing process, however, is only dependent on CR3 (59). On the other hand, it suggested that inhibition of NETs release might contribute to the treatment of patients with IPA (60). They found that in the IPA model, mice lacking PAD4 had a lower fungal burden in their lungs and less acute lung injury. This indicates that NETs release causes tissue damage and impairs fungal clearance in IPA mouse models (60).

### C. neoformans

**Figure 1:**
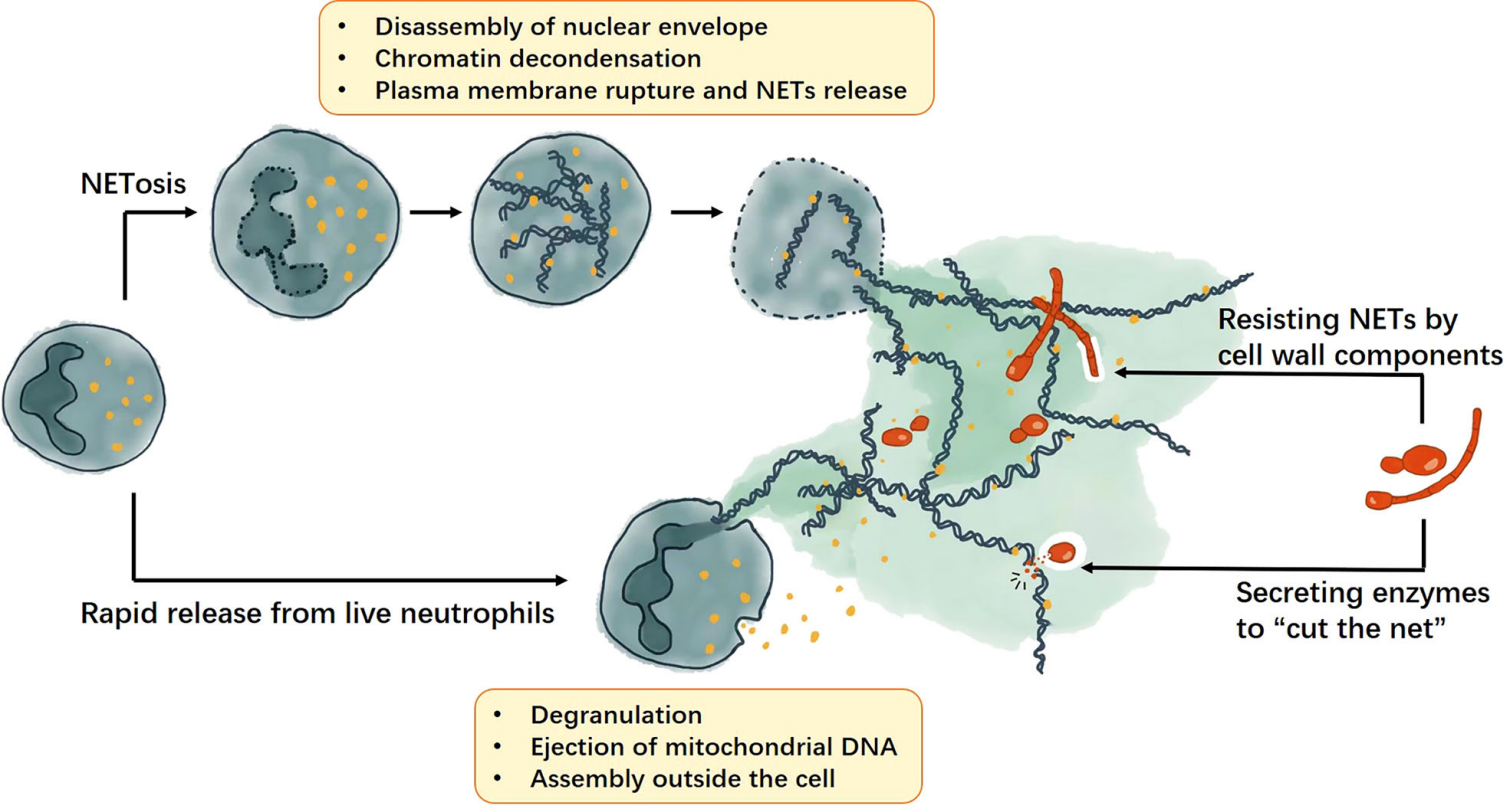
The formation of NETs and the fungal strategies against NETs. NETs can be produced though NETosis or rapid release from live cells. The NETosis process includes disassembly of nuclear envelope, chromatin decondensation, plasma membrane rupture and NETs release; whereas rapid release from live cells require cell degranulation and ejection of mitochondrial DNA, then the NETs will be assembled outside the cell. For fungi, they can resist NETs by cell wall components and secrete enzymes to help them escape the NETs.

*C. neoformans* is also an opportunistic fungal pathogen with a small-size yeast form and a unique polysaccharide capsule. It is one of the most common pathogens for meningitis (61). Polysaccharide capsules are considered to be a key virulence factor (62). It comprises approximately 88% glucuronoxylomannan (GXM),10% GXMGal, and 2% mannoproteins (63, 64). Although wild-type *C. neoformans* and its GXM do not induce NETs, NET-enriched supernatants induced by a mutant acapsular strain exhibit fungicidal activity against wild-type strains. (34) (**Figure 1**)

## Fungal strategies against NETs

Hosts can kill fungi by producing NETs, but the fungi won’t stand still. Fungal defense strategies against NETs are varied and can be generally divided into two categories: modulation of NETs formation and escape from NETs. On the one hand, fungi can modulate the formation of NETs through their own components, thus resisting or even inhibiting the fungicidal effects of NETs. This phenomenon is common in *Aspergillus* spp., *C. albicans*, and *C. neoformans*. On the other hand, fungi can release active proteins, usually enzymes that target nuclear acids, to help themselves escape NETs.

### Modulation of NETs formation

*Aspergillus* spp. can use galactosaminogalactan (GAG) to enhance resistance to NETs. GAG is an exopolysaccharide produced by *A. fumigatus* and is associated with adherence and complete virulence. Enhancing GAG in less pathogenic *A. nidulans* at the genetic level can increase its virulence and resistance to NADPH oxidase-dependent NETs *in vitro*. It indicates that cell wall-bound GAG enhances virulence through mediating resistance to NETs (65). CcpA is another important protein to reduce recognition by the innate immune system. Lacking of CcpA causes higher activation of neutrophils and speeds up the oxidative burst progress, and *A. fumigatus* Δ*ccpA* conidia shows highly attenuated virulence even in immunosuppressed mice (66).

*C. albicans* also has its unique ways to resist NETs. *C. albicans* biofilms of clinical isolates uniformly impair NETs release at different depths and architectures (67). Another way for *C. albicans* to modulate NETs formation is by arresting proteinous components of NETs, including elastase, myeloperoxidase, lactotransferrin, and histones. These NETs components are involved in cell surface contact with *C. albicans*. Adhesins on the surface of *C. albicans*, such as the agglutinin-like sequence protein family Als3, can adsorb NETs proteins and increase the pathogen’s potency in host cell destruction, suggesting that the efficiency of fungal entrapment might be altered (68).

*C. neoformans* appears to be quite “invisible” to NETs. The fungus itself and its major capsular polysaccharide glucuronoxylomannan (GXM) do not trigger NETs formation. Moreover, both inhibit the production of PMA-induced NETs. In addition, both GXM and GXMGal block the production of ROS through PMA-activated neutrophils (34).

### Escape from NETs

*C. albicans* and *C. glabrata* can escape from being trapped in NETs through their 3’-nucleotidase/nuclease (3’NT/NU) activity. 3’NT/NU is an ectonucleotidase that hydrolyze AMP and nucleic acids. When NETs trap *Candida* cells, the cells promote NETs disruption and this process can be blocked by 3’NT/NU inhibitor ammonium tetrathiomolybdate (69).

Besides, *C. albicans* can escape NETs by secreting DNase. Strains that secrete more DNase showed greater resistance to neutrophil killing. And the antifungal activity of neutrophils decreases significantly after NETs being degraded by exogenous DNase I or catalase (70).

## Outlook

It is like a seesaw battle between NETs and pathogenic fungi. NETs are activated when fungi invade the host, trapping the fungi and killing them. The fungi, in turn, find their ways to resist NETs’ fungicidal effect or escape traps.

Research on NETs has been a hotspot in recent years. NETs protect the host from infections by killing pathogens including bacteria, fungi, viruses, and parasites. However, in addition to antimicrobial effects, excess NETs increase pro-inflammatory responses and cause damage to surrounding tissues, which has negative effects in many infectious and non-infectious diseases. For example, dysfunction of NETs can damage host tissues, promote the development of autoimmunity and thrombosis (71). NETs are also involved in nearly all the inflammation-related diseases, including systemic lupus erythematosus (72), rheumatoid arthritis (72), atherosclerosis (73), diabetes (74), asthma (75), tumors (75), and wound healing (76). Since its discovery, people have been enthusiastic about this field, and there are still many unknowns to explore. Taking the antifungal activity as an example, the role of NETs in fungal infections is still unclear. What are the molecular mechanisms underlying the induction, formation, and antifungal processes of NETs? Why do different fungi, or even different strains of the same species induce NETs differently?

As our understanding of NETs’ underlying mechanisms increases, it may provide a useful tool for diagnosis and treatment of related diseases. On the one hand, we can identify new targets and design drugs that enhance the antifungal ability of NETs without causing tissue damage. On the other hand, ideal NETs release blockers may be discovered and used to avoid tissue damage without compromising antimicrobial effects. We can also use synergists to reduce immune escape or resistance of fungi to NETs. Another interesting idea is that, according to a recent study (77), extracellular traps can be trained as a memory response. There may one day be a vaccine to help increase the antimicrobial function of NETs in high-risk populations.

## Author contributions

All authors listed have made direct and intellectual contribution to the work and approved it for publication.

## Funding

This study was supported by the National Natural Science Foundation of China (81772124), the Shanghai Science and Technology Commission “Science and Technology Innovation Action Plan” biomedical science and technology support special project (20S11902900) and the biosafety program (20SWAQX29-1-6).

## Conflict of interest

The authors declare that the research was conducted in the absence of any commercial or financial relationships that could be construed as a potential conflict of interest.

## Publisher’s note

All claims expressed in this article are solely those of the authors and do not necessarily represent those of their affiliated organizations, or those of the publisher, the editors and the reviewers. Any product that may be evaluated in this article, or claim that may be made by its manufacturer, is not guaranteed or endorsed by the publisher.
